# Mathematical modeling and numerical simulation of arterial dissection based on a novel surgeon’s view

**DOI:** 10.1007/s10237-023-01753-y

**Published:** 2023-08-08

**Authors:** Meisam Soleimani, Rohan Deo, Blaz Hudobivnik, Reza Poyanmehr, Axel Haverich, Peter Wriggers

**Affiliations:** 1 Institute of Continuum Mechanics, Leibniz University, Hannover, Germany; 2 Klinik für Herz-, Thorax-, Transplantations- und Gefäßchirurgie, Medical School, Hannover, Germany

**Keywords:** Dissection, Vasa vasorum, Phase-field modeling, Atherosclerosis, Finite element method

## Abstract

This paper presents a mathematical model for arterial dissection based on a novel hypothesis proposed by a surgeon, Axel Haverich, see Haverich (Circulation 135(3):205–207, 2017. 10.1161/circulationaha.116.025407). In an attempt and based on clinical observations, he explained how three different arterial diseases, namely atherosclerosis, aneurysm and dissection have the same root in malfunctioning Vasa Vasorums (VVs) which are micro capillaries responsible for artery wall nourishment. The authors already proposed a mathematical framework for the modeling of atherosclerosis which is the thickening of the artery walls due to an inflammatory response to VVs dysfunction. A multiphysics model based on a phase-field approach coupled with mechanical deformation was proposed for this purpose. The kinematics of mechanical deformation was described using finite strain theory. The entire model is three-dimensional and fully based on a macroscopic continuum description. The objective here is to extend that model by incorporating a damage mechanism in order to capture the tearing (rupture) in the artery wall as a result of micro-injuries in VV. Unlike the existing damage-based model of the dissection in the literature, here the damage is driven by the internal bleeding (hematoma) rather than purely mechanical external loading. The numerical implementation is carried out using finite element method (FEM).

## Introduction

Arteries are classified into two distinct types: elastic arteries and muscular arteries. Elastic arteries, such as the aorta and the common carotid and iliac arteries, have larger dimensions than muscular arteries, for example, the coronaries, the femoral and renal arteries Humphrey (1995). A healthy artery wall mainly consists of three layers: the intima (the innermost layer), the media (the middle layer), and the adventitia (the outermost layer). The media gives the most significant mechanical strength to the artery wall in both the longitudinal and circumferential directions due to its structural arrangement, which is similar to laminate composites MacLean et al. (1999). In the artery wall, the two families of collagen fibers are almost equally distributed concerning the axis of the artery, with the fiber orientation in the adventitia and the media closer to the axial direction and the circumferential direction, respectively Schriefl et al. (2015). In addition to these layers, a vascular network of tiny blood vessels known as Vasa Vasorum (VV) is present in the large or medium-sized arteries for the complete nourishment of the arterial wall.

Arterial dissection is described as delamination between the different layers as well as the separation of the laminated structure of the artery wall. The mechanical phenomenon of arterial dissection can be split into two separate processes, namely initiation and propagation. According to Rajagopal et al. Rajagopal et al. (2007), the commencement of the aortic dissection can be triggered due to high systolic blood pressure, whereas propagation is influenced by pulse pressure and heart rate. For example, in some types of aortic dissections, the propagation of the initial tear in the intima permits pathological blood within layers of the media Gasser and Holzapfel (2006); Sommer et al. (2008). This pressurized blood expands the split and may create an additional passage known as the false lumen by compressing the true lumen Chen et al. (2013). It might be lethal since it causes a decrease in blood flow to vital arteries. Another closely related appearance of dissection is an intramural hemorrhage/hematoma (IMH) Thubrikar and Agali (1999); Khan and Nair (2002). In IMH, the hematoma initiates the delamination of the different layers, increases in size plus expansion, and ultimately erupts into the true lumen. There is yet no consensus on whether an IMH is a separate entity, or if it is a preliminary stage of arterial dissection (Which came first, the chicken or the egg?). However, it has been identified that the conversion rate of an IMH into an aortic dissection, with its above-mentioned complications, is up to 88–90% Alomari et al. (2014).

Numerous investigations on arterial dissection imply and endorse the intimal tear and IMH as incitement to the propagation of dissection. Based on several cardiovascular surgeries, Axel Haverich established a unified hypothesis, which describes atherosclerosis as a microvascular disease triggered by VV occlusion inside the outer layer of the arteries (adventitia) that culminates with arterial functional impairment Haverich (2017). As mentioned earlier, VV is required to provide nutrients to artery walls in large- and medium-sized arteries. However, the obstruction of VV by viruses, bacteria, and tiny dust particles, which are most likely induced due to risk factors such as hypertension, smoking and age can disrupt the nutrition supply to the wall tissues. Consequently, VV experiences various ischemic processes that lead to inflammation mechanisms. Thus, elevated systemic inflammation would be the prime initiation of arterial dissection. During his cardiac surgeries, Axel Haverich Haverich (2017) noticed that non-atherosclerotic large and medium arteries possess no VV in the adventitial layer, implying no risk of wall ischemic processes. The mathematical model established in the present work is primarily based on the hypothesis presented in Haverich (2017).

In early experimental research, Tam et al. Tam et al. (1998) studied the tear propagation driven by pressure in porcine thoracic aortas under static conditions. It showed an inverse relationship between the propagation pressure and the initial depth of the tear. Furthermore, Sommer et al. conducted peeling tests on the specimens of human aortic media and observed variations in dissection energy in circumferential and axial directions Sommer et al. (2008). Pasta et al. performed peel tests on the aneurysm aorta due to the increasing possibility of dissecting the aneurysm Pasta et al. (2012). Likewise, direct peel and tension tests were also implemented on the left anterior descending coronary artery Wang et al. (2013) and a carotid artery Tong et al. (2011) to investigate dissection properties. According to a recent experimental study Kozuń et al. (2018), researchers investigated that the evolution of atherosclerosis decreases artery wall resistance against dissection. Another study from the same research group observed that the dissection process differed between the ascending aorta and ascending aneurysmal aorta Kozuń et al. (2019). Haslach et al. carried out pressure inflation experiments on aortic ring specimens with different orientations of notches Haslach et al. (2018). This work concluded that shear rupture is driving aortic dissection as well as that shear tests, rather than tensile strength tests, may provide effective evaluations for the strength of the artery wall.

Besides clinical observation, arterial dissection has always been a fascinating subject among researchers in the field of computational biomechanics. Gasser and Holzapfel presented the first numerical study of peeling tests in the context of the partition of unity finite element method (FEM), coupled with cohesive crack theory, to examine the dissection propagation of aortic media Gasser and Holzapfel (2006). Ferrara and Pandolfi employed cohesive zone modeling based on traction-separation law to simulate peeling processes Ferrara and Pandolfi (2010). Both contributions adapted the Holzapfel-Gasser-Ogden material to account for the anisotropic hyperelasticity of the artery wall. Wang et al. investigated the initiation and propagation of the dissection in the framework of the extended FEM and created the residually stressed artery model Wang et al. (2016, 2017).

Based on the hypothesis of Humphrey Humphrey (2012), the semi-analytical and FEM-based continuum methods were used by Roccabianca et al. Roccabianca et al. (2013) to investigate the influence of pooled glycosaminoglycans (GAGs). The findings demonstrate a substantial intramural stress concentration around the accumulation of GAGs as a result of intra-lamellar swelling pressure. Furthermore, particle-based computational studies Ahmadzadeh et al. (2019) examined the initiation and progression of intra-lamellar (medial) dissection under the impact of pooled GAGs. In a recent study Rolf-Pissarczyk et al. (2021), a discrete fiber dispersion model was employed to analyze the deterioration of interlamellar elastic fibers during delamination of the aorta.

In a recent phase-field modeling approach Ban et al. (2021, 2022), Ban and colleagues investigated the aortic dissection to examine the correlation between the pressure-volume curve and the intramural fluid that originates and propagates intramural delamination, employing the microstructure suggested by histology. In particular, they simulated the experimental investigations conducted by Roach and Song Roach and Song (1994) as well as verified the power law behavior indicated by Yu et al. Yu et al. (2020). Gültekin et al. Gültekin et al. (2016) employed a phase-field model to simulate peeling and simple shear experiments performed on the aortic wall. Phenomenologically equivalent to previous research, an anisotropic failure criterion based on the fracture energy of the components (the ground matrix and the collagen fibers) of the material model has been implemented in this model. In a recent research work Gültekin et al. (2019), Gültekin et al. extended the previous analysis by adapting a similar crack phase-field approach and came to the same conclusions as Haslach et al. (2018) that aortic dissection is characterized by an in-plane shear-driven process.

The outcome of the mathematical models can be validated against clinical medical data. For example, in the case of aortic dissection, CT or MRI images are pretty usable. High-resolution images allow precise descriptions of aortic wall thickness, constitution, morphology and wall configuration Ko et al. (2021); Murillo et al. (2021). They can be employed in diagnosing aortic pathologies of all kinds, especially aortic dissections and IMH with a sensitivity of as high as 96% 2014 ESC guidelines on the diagnosis and treatment of aortic diseases (2014).

In this contribution, the authors extend the multiphysics model presented in Soleimani et al. (2021) to predict the emergence of dissection by introducing a specific damage model that captures the tearing in the arterial wall. The existing damage-based models of dissection in the literature are identical to classical fracture mechanics problems in which an external mechanical loading (here blood pressure in the lumen) leads to crack propagation in a notched specimen. The distinction between the presented phase-field model of dissection and those available in the literature is that here the damage is triggered and driven by IMH in addition to the presence of mechanical loading due to lumen blood pressure. In this sense, the problem is similar to hydraulic fracture (hydrofracking) Mauthe and Miehe (2017).

**Fig. 1:**
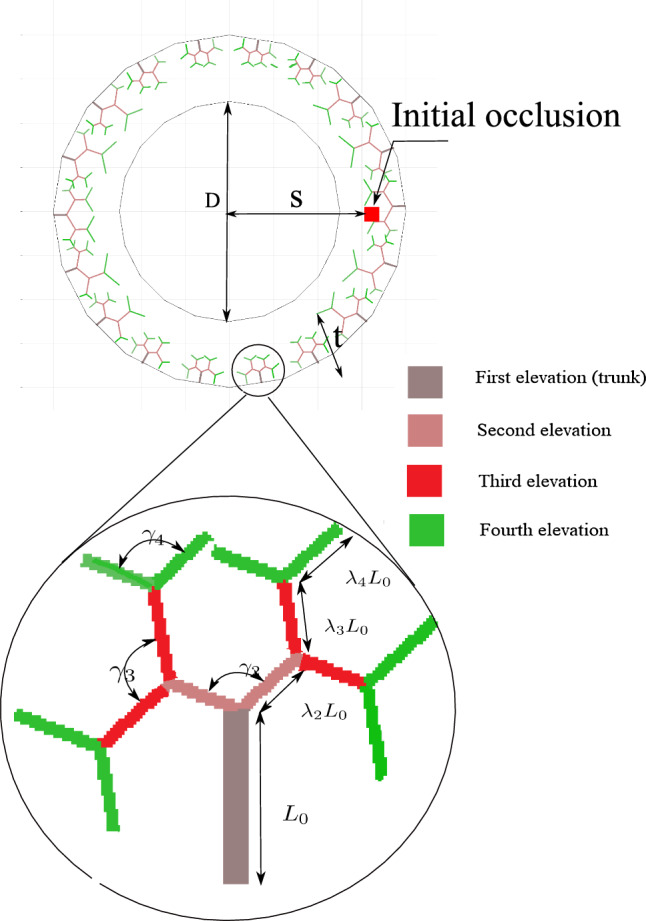
Geometrical representation of VV using tree fractal concept and also initial occlusion, from Soleimani et al. (2021)

## Mathematical modeling of arterial dissection

**Fig. 2:**
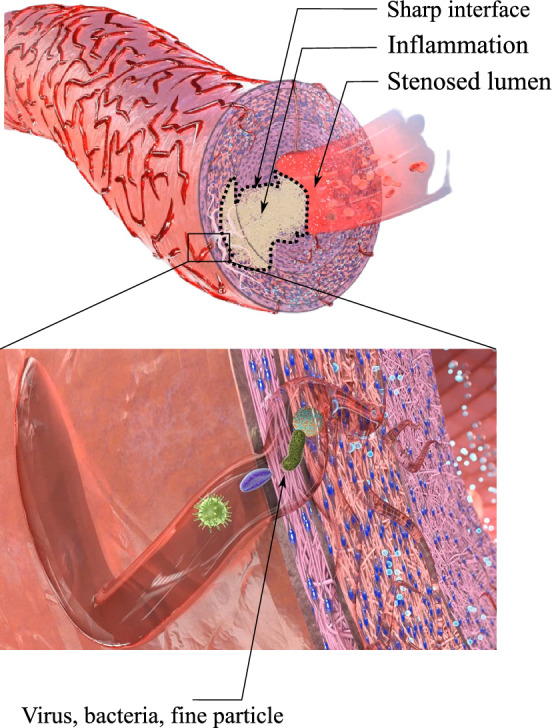
Inflammation as a response to the VV occlusion due to viruses, bacteria and fine particle, from Soleimani et al. (2021)

**Fig. 3:**
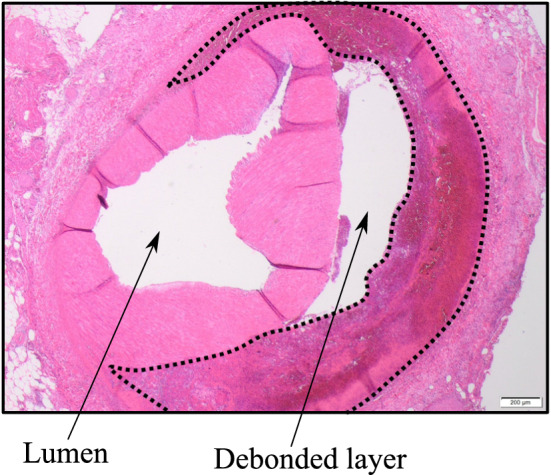
Histopathology of IMH in an arterial vessel. Hematoxylin and Eosin (H&E) stained artery shows fresh extravasation of erythrocytes into the vessel wall (area in the dotted line with subtotal stenosis of the vascular lumen

A coupled multi-physics approach is used to characterize the progression of atherosclerosis as well as the dissection of the arterial wall. Firstly, the mechanical deformation captured by the displacement field $\varvec{u}$ is governed by the conservation of linear momentum. Secondly, the nutrient concentration is determined by the scalar variable *c*, indicating the availability of the nutrients, and its transportation follows a classical diffusion–reaction equation. Lastly, the phase-field variables $\phi$ and *d* represent the inflammation (overgrowth) and rupture (damage) of the arterial tissue, respectively, using Allen-Cahn type Allen and Cahn (1979) phase-field modeling. The following “assumptions" and “hypotheses" imply a physically significant correlation between these diverse fields:

- **Assumptions:**
  1. To reduce the complexity, we consider the multi-layered structure as a single-layer structure with similar material properties and orientations of the collagen fibers for all layers.
  2. The blood flowing in the lumen delivers the nutrients to tissues near the intima layer via diffusion. Moreover, the tissues closer to the adventitia layer are nourished by VV for a sufficient supply of nutrition to the entire arterial wall. As shown in Fig. 1, the 2D structure of VVs, which initiates on the exterior part of the artery wall and permeates up to the central portion of the wall, is generated stochastically utilizing an open-source MATLAB code Tyutyunnikov (2006). Figure 1 also depicts the parameters that significantly influence the geometrical shape of VVs.
  3. The impact of blood flow is incorporated through physical boundary conditions, instead of modeling in an explicit manner. Specifically, the interfaces interacting directly with the blood are preset to have the maximum nutrient concentration. Moreover, the innermost surface of the wall is subjected to the systolic mean pressure exerted by the blood flow in the lumen.
  4. For simplification, we assume that all of the cells in the arterial wall consume nutrients at a uniform rate. Likewise, the nutrient’s diffusivity across the wall is kept constant before initiating inflammation and is assumed to reduce as the inflammation progresses due to a denser texture of the tissue.
  5. A finite strain theory including a multiplicative decomposition of the deformation gradient is employed to formulate the large mechanical deformation of the wall induced by the overgrowth.
- **Hypotheses:**
  In case of atherosclerosis
  1. As shown in Fig. 2, the occlusion of VV results in nutrient deficiency inside the outer layer of the wall, leading to the emergence and evolution of inflammation. Mathematically, a threshold criterion for the nutrient is established below that inflammation occurs, connecting the nutrition transport equation with the inflammation phase-field equation.
  2. The development of the lesion is in the direction of maximum change of nutrients. From a mathematical point of view, the sharp interface between the inflammatory and healthy regions, as illustrated in Fig. 2, is interpreted as the lesion boundary. The advection of this boundary is in the direction of the nutrient gradient (maximum nutrient change).
  3. The overgrowth, is directly proportional to inflammation, establishing the connection between the phase-field variable and mechanical deformation.
  In case of dissection
  1. As shown in Fig. 2, the occlusion of VV results in an IMH which means simply bleeding. The function of the hematoma in dissection is similar to that of inflammation in atherosclerosis and hence it is described using a phase-field equation.
  2. The development of the lesion is driven by blood perfusion. The sharp interface between the hematoma and healthy regions, as illustrated in Fig. 2, is interpreted as the lesion boundary. The advection of this boundary depends on how the damaged (ruptured) zone evolves, see Fig. 3.
  3. The overgrowth, is directly proportional to hematoma development, establishing the connection between the phase-field variable and mechanical deformation.

The two aforementioned hypotheses are in line with the objective of this work, namely establishing a mathematical model that unifies both atherosclerosis and dissection in the same framework. As stated, both pathologies originate from dysfunctioning arterial capillaries (VVs). Rupturing VV as the leading cause and pathomechanism of IMH has been described before Alomari et al. (2014). Though risk factors like arterial hypertension or atherosclerosis are already identified, the initiating event, which leads to the formation of IMH remains unknown Alomari et al. (2014); Baikoussis et al. (2009). Only and certainly not a satisfactory explanation so far is “spontaneously”. However, the hypothesis proposed here has good supporting and already published evidence regarding VV dysfunction and disease initiation Haverich (2017). In fact, Köster Köster (1876) was one of the first, who observed and described, that “obstruction” of VV leads to necrosis of the tunica media and potential development of atherosclerosis, ultimately leading to rupture of VV. As a cause of obstruction, syphilitic aortitis has been described before. Spirochetes have been repeatedly found inside the aortic wall resulting in inflammatory obstruction of VV resulting in wall ischemia and consecutively necrosis, aneurysm formation and rupture O’Regan (2002); Stone et al. (2015). Not only spirochates have been found in VV, RNA and DNA analysis revealed other bacterial and viral components supporting our hypothesis of infection-triggered obstruction of the VV. Another significant pathway of intimal bleeding is angiogenesis. Angiogenesis is the process of blood vessels sprouting from preexisting vessels. Neovessel formation is triggered primarily in response to hypoxia and/ or inflammatory signals. Neovessels, in general, are inherently immature, fragile and leaky Jeziorska and Woolley (1999); Dunmore et al. (2007).

Based on the assumptions and hypotheses stated above, one can now formulate the governing equations for the multiphysics problem at hand.

### Mechanical equilibrium equation

As indicated in assumption 5, the idea for the formulation of overgrowth originates from multiplicative finite plasticity, in which the total deformation gradient is split through the multiplicative decomposition according to

1 $$\begin{aligned} \varvec{F}= \varvec{F}_e \varvec{F}_g, \end{aligned}$$

where $\varvec{F}_e$ corresponds to the elastic part of the mechanical deformation and $\varvec{F}_g$ captures the overgrowth due to inflammation. The spatial gradient of the displacement field $\varvec{u}$ can be utilized to compute the deformation gradient $\varvec{F}$ such that

2 $$\begin{aligned} \varvec{F}=(\varvec{I}-\nabla \varvec{u})^{-1}, \end{aligned}$$

in which $\nabla$ refers to the gradient operator in the spatial coordinates.

The conservation of linear momentum for the artery in the spatial coordinates using the assumption of quasistatic process and body forces equal to zero can be given by

3 $$\begin{aligned} \nabla \cdot \varvec{\sigma }=0, \end{aligned}$$

in which $\nabla \cdot$ denotes the spatial divergence operator and $\varvec{\sigma }$ is the Cauchy stress tensor. Since atherosclerosis is considered a slow process, the so-called non-compliant terms pertaining to the growth process are neglected in the conservation equation of linear momentum Goriely (2017).

Furthermore, one needs to assume a free energy function $\Psi$ (constitutive relationship) from which the stress tensor can be computed as follows

4 $$\begin{aligned} \varvec{\sigma }=\frac{1}{J_e}\frac{\partial \Psi }{\partial \varvec{F}_e } \varvec{F}^T_e, \end{aligned}$$

where $J_e$ is the determinant of the elastic deformation gradient ($J_e=\text {Det}(\varvec{F}_e)$). A nearly incompressible and isotropic hyperelastic neo-Hookean free energy function is adopted according to

5 $$\begin{aligned} \Psi =\Psi _{iso }+\Psi _{vol }=\underbrace{\frac{\mu }{2}(\hat{I}_{1e}-3)}_{\begin{array}{c} \text {Isochoric energy} \\ \text {contribution} \end{array}}+\underbrace{\frac{\nu \mu }{(1-2\nu )}(J_e-1)^2-\mu \ \text {Log}J_e}_{\begin{array}{c} \text {Volumetric energy} \\ \text {contribution} \end{array}}, \end{aligned}$$

where $\mu$ and $\nu$ represent the constant material parameters. Additionally, $\hat{I}_{1e}$ is the first invariant of the isochoric right Cauchy-Green tensor defined as $\hat{\varvec{C}}_e=J^{-\frac{2}{3}}_e\varvec{F}_e^T \varvec{F}_e$ and can be computed using

6 $$\begin{aligned} \hat{I}_{1}=\text {Tr}(\hat{\varvec{C}_e}). \end{aligned}$$

### Nutrient transport equation

The nutrient transport in the arterial wall is considered to obey the classical diffusion–reaction equation

7 $$\begin{aligned} \nabla \cdot (D\nabla c)-R_c=0, \end{aligned}$$

where *c* denotes the nutrient concentration and *D* represents the diffusivity coefficient. Further, $R_c$ defines the rate of nutrient consumption in cells, which is the sink term in this equation. As mentioned in assumption 4, $R_c$ is kept constant and uniform, while *D* reduces due to inflammation. For simplicity, a linear equation based on the inflammation state $\phi$ as shown below can be used to model the transition of the diffusivity coefficient

8 $$\begin{aligned} D=\phi D_{min } + (1-\phi )D_{max }, \end{aligned}$$

where $D_{max }$ and $D_{min }$ are the diffusivity in the healthy and inflammatory arterial tissue, respectively.

It should be noted that the time-dependent part is omitted from the nutrient transport equation because the diffusion process has a substantially shorter time scale than the inflammation process.

### Inflammation phase-field equation

A phase-field variable termed $\phi$ is used to determine whether or not a specific region of tissue has undergone the inflammation process. From a mathematical standpoint, $\phi$ is a binary indicator bounded in the interval [0,1], expressing the status of the inflammatory phase at the point of interest. In specific, $\phi =0$ indicates no inflammation, whereas $\phi =1$ signifies the occurrence of inflammation. The boundary of inflammatory cells is characterized by a sharp interface between 0 and 1.

In order to capture the interface between the inflammatory and the surrounding healthy tissue, the Allen-Cahn type phase-field model is utilized as follows

9 $$\underbrace {{\dot{\phi }}}_{{{\text{Phase evolution}}\;{\text{in pseudo time}}}} = \underbrace {{ - M\frac{{\partial f(\phi )}}{{\partial \phi }}}}_{{{\text{Bulk contribution}}}} + \underbrace {{\epsilon ^{2} \nabla ^{2} \phi }}_{{{\text{Sharp interface contribution}}}} + \underbrace {{S_{\phi } (c,d),}}_{{{\text{Driver (source) of phase - field}}}}$$

where the parameters *M* and $\epsilon$ govern the energy jump and the width of the interface between the phases, respectively. The function $f(\phi$) describes the barrier that must be overcome for a phase transformation and is commonly given by

10 $$\begin{aligned} f(\phi )=16M \phi ^2(1-\phi )^2, \end{aligned}$$

in which $M=f({1}/{2})$ is the local maximum value of the function between the two cells at $\phi =1$ and $\phi =0$.

Moreover, $S_{\phi }(c,d)$ signifies the source term of the equation. It is, indeed, the driver of the phase-field. Based on hypotheses 1 and 2, a physically meaningful expression is employed to represent the function $S_{\phi }(c,d)$ as

11 $$\begin{aligned} S_{\phi }(c,d)=R_s \, c\, d, \end{aligned}$$

where the parameter $R_s$ controls the magnitude of the source term. A clear interpretation exists for equation (11) and hence the hematoma growth depends on two things: The damaged (ruptured) region captured by *d* and the blood availability represented by *c*. In fact, the ruptured region provides room for blood perfusion. A linear dependency is, of course, the simplest assumption but not the only possible model.

One may refer to Soleimani et al. (2021) for more details regarding the choice of this specific form of the source function as well as the phase-field model.

In order to invoke the third hypothesis, the inflammation state $\phi$ should be linked to the mechanical part of the growth tensor $\varvec{F}_g$. By the assumption of isotropic overgrowth, $\varvec{F}_g$ can be expressed by a scalar $\alpha$ and the identity tensor $\varvec{I}$, as shown below

12 $$\begin{aligned} \varvec{F}_g=(1+\alpha ) \varvec{I}. \end{aligned}$$

The scalar parameter $\alpha$ captures the overgrowth. It is implemented as an internal variable and set to zero initially. The velocity gradient related to the rate of growth tensor can be calculated by

13 $$\begin{aligned} {\varvec{L}}_g=\dot{\varvec{F}}_g \varvec{F}_g^{-1}=\frac{\dot{\alpha }}{1+\alpha }\varvec{I}, \end{aligned}$$

in which the dot denotes the time derivative. As postulated in Soleimani et al. (2021), one can linearly connect the overgrowth magnitude to the rate of hematoma state (similar to the inflammation state in the case of atherosclerosis) as follows

14 $$\begin{aligned} \frac{\dot{\alpha }}{1+\alpha }=k_g \dot{\phi }, \end{aligned}$$

where $k_g$ is a proportionality coefficient, chosen as the model parameter.

### Damage (crack) phase-field equation

In order to capture the rupture within a particular region, the phase-field variable denoted as *d* is utilized. The central idea of this approach is to regularize a sharp crack interface by a diffusive crack topology. The phase-field variable is a scalar-valued function in the interval [0,1], characterizing for $d = 0$ the intact (solid) state and for $d = 1$ the fully cracked (ruptured) state of the tissue. It is important to note that the phase-field variable is formulated in the reference configuration.

Following the arguments outlined in Miehe et al. (2010), the sharp crack interface is considered to be governed by

15 $$\begin{aligned} d - l^2 \nabla ^2d=0, \end{aligned}$$

where the parameter *l* is the width of the crack zone. It corresponds to the crack surface energy defined by the equation

16 $$\begin{aligned} \gamma (d,\nabla d)=\frac{d^2}{2} +\frac{l^2}{2} \nabla d \cdot \nabla d. \end{aligned}$$

The rupture of the artery is an irreversible process from a physical point of view. It results from a local state of tension which leads to the propagation of a crack. Therefore, we decompose the volumetric part of the free energy ($\Psi _{vol}$) into a tensile part and fracture-insensitive compressive part. Hence the volumetric part of free energy in equation (5) is replaced with

17 $$\begin{aligned} (1-H_J) g(d) \Psi _{vol } + H_J \Psi _{vol }, \end{aligned}$$

in which

18 $$\begin{aligned} H_J = {\left\{ \begin{array}{ll} 1&{}\text {if}\, J_e\le 1\\ 0 &{}\text {if}\, J_e> 1 \end{array}\right. }, \end{aligned}$$

and the degradation function *g*(*d*) is defined as

19 $$\begin{aligned} g(d)=(1-d)^2+d\ d_{min }, \end{aligned}$$

in which $d_{min }$ is a numerical parameter close to zero in order to avoid singularity when *d* approaches 1. This function is monotonically decreasing if the damage variable *d* increases gradually.

One can see that the volumetric part of the energy is degraded if it is in a tensile state. Furthermore, the isochoric part of the energy is always degraded irrespective of the tensile/compressive state. Hence, $\Psi _{iso }$ in equation (5) is replaced with

20 $$\begin{aligned} g(d) \Psi _{iso }. \end{aligned}$$

Similar to equation (9), the Allen-Cahn type equation for the damage field can be written as

21 $$\underbrace {{\eta _{d} \dot{d}}}_{{{\text{damage evolutionin pseudo time}}}} = \underbrace {{-d + l^{2} \nabla ^{2} d}}_{{\begin{array}{*{20}c} {{\text{surface energy}}} \\ \end{array} }} + \underbrace {{S_{d} (\varvec{u},d),}}_{{{\text{Driver (source) of damage field}}}}{\text{ }}$$

in which $\eta _d$ is an artificial viscosity and *l* refers to the damage length-scale parameter. Additionally, the damage source term, $S_d(\varvec{u},d)$, is driven by the mechanical part and can be written as

22 $$\begin{aligned} S_d(\varvec{u},d) = -\frac{\partial g(d)}{\partial d} \frac{ \Psi _{max }}{\Psi _{cri }/l}. \end{aligned}$$

Note that the reversibility of damage (healing) is prevented by treating $\Psi _{max }$ as a history variable meaning that the maximum value of the free energy appearing in the course of the time is used. In practice, when it comes to numerical implementation, the local energy density $\Psi$ is compared with that of the previous time step, and then the maximum of these two is stored as the history variable. In mathematical terms

23 $$\begin{aligned} \Psi _{max }=\text {MAX}[\Psi ^{n+1},\Psi ^{n}], \end{aligned}$$

in which the superscripts *n* and $n+1$ refers to the previous and current time steps, respectively. Equation (23) ensures that the damage variable *d* is either increasing (during loading) or frozen (during unloading).

## Numerical implementation using FEM

In order to implement the equations discussed in the previous section, we adopt a standard Galerkin FEM. Considering the week form of equations (3), (7), (9) and (21), one can construct a Lagrangian $\mathcal {L}$ as a function of primary variables $\varvec{u}$, *c*, $\phi$ and *d*. Stationary conditions $\delta \mathcal {L}=0$ leads to the desired weak form

24 $$\begin{aligned} \begin{aligned} \delta \mathcal {L}(\varvec{u},c,\phi ,d) =&\int _{\mathcal {B}} (\nabla \cdot \varvec{\sigma })\cdot \delta \varvec{u} \ dv \\&\quad +K_c \int _{\mathcal {B}} [D \nabla ^2 c ) \delta c+R_c \delta c] dv\\&\quad +K_{\phi }\int _{\mathcal {B}} [(\epsilon ^2\nabla ^2 \phi ) \delta \phi +M \frac{\partial f(\phi )}{\partial \phi }\delta \phi - S_{\phi }(c,d)\delta \phi +\dot{\phi }\ \delta \phi ]\ dv \\&\quad +K_d \int _{\mathcal {B}} [d \ \delta d - l^2 \nabla ^2d \ \delta d-S_d(\varvec{u},d)\delta d+\eta _d \dot{d} \ \delta d] \ dv=0, \end{aligned} \end{aligned}$$

in which $K_{\phi }$, $K_c$ and $K_d$ are the numerical parameters employed to get a suitable condition number of the multi-field global stiffness matrix. Their selection significantly impacts the performance of the monolithic approach in solving the multi-field problem.

**Fig. 4:**
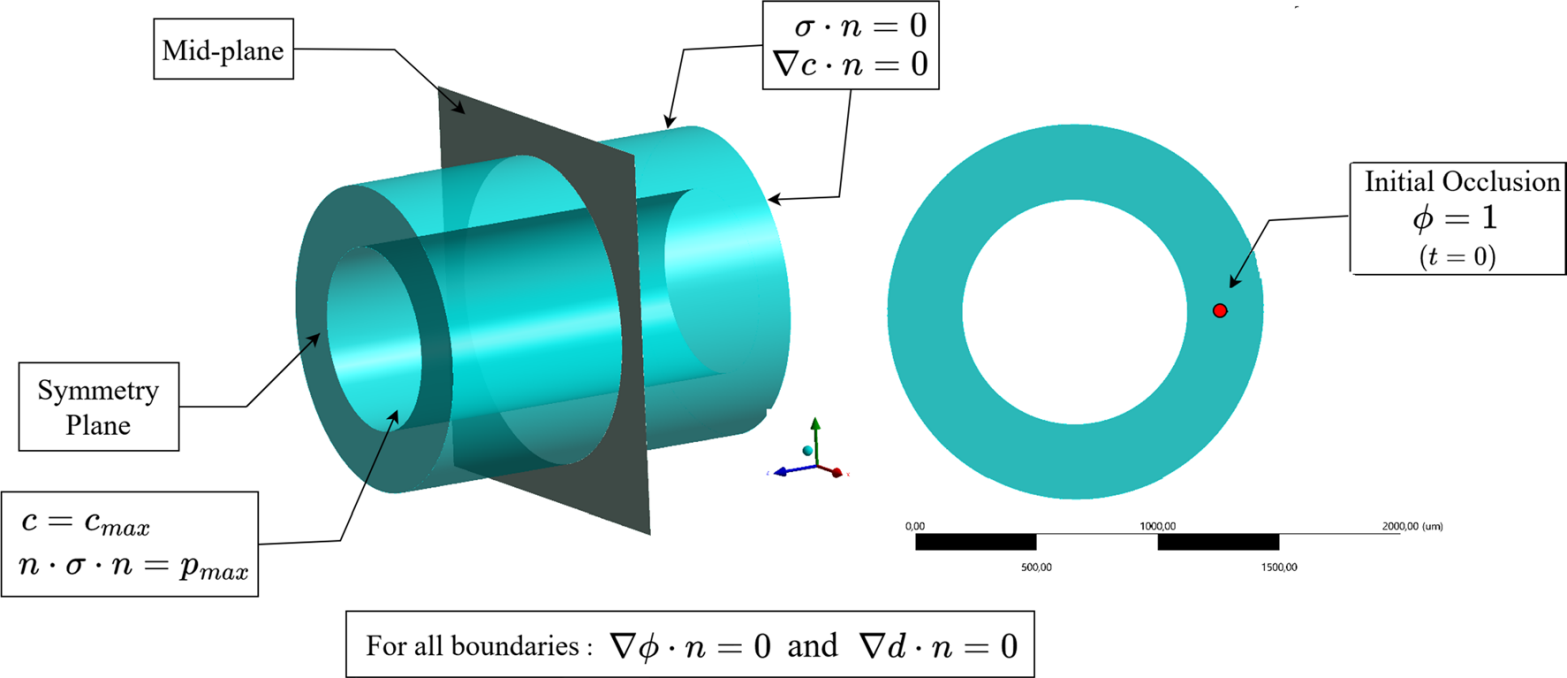
Geometrical model of the artery with boundary conditions

After the formulation of the weak form, integration by parts is applied to the integrals in equation (24), which includes now the boundary terms specified on the boundary ${\partial {\mathcal{B}}}$. In addition, a backward (implicit) Euler scheme is used to approximate the time derivative terms ($\dot{\phi }$ and $\dot{d}$). In this regard, the superscript $t-\Delta t$ refers to the previous time step. As a result, the weak form in equation (24) leads to

25 $$\begin{aligned} \begin{aligned} \delta \mathcal {L}(\varvec{u},c,\phi ,d) = &\int _{\mathcal {B}} \varvec{\sigma }: \nabla ^{sym } \delta \varvec{u} \ dv -\int _{\partial \mathcal {B}} \varvec{t} \cdot \delta \varvec{u} \ ds \\&+K_c \int _{\mathcal {B}} [D \nabla c \cdot \delta \nabla c\ +R_c \delta c] \ dv -K_c \int _{\partial \mathcal {B}} D \nabla c\cdot \varvec{n} \ \delta c\ ds \\&+K_{\phi }\int _{\mathcal {B}}[\epsilon ^2\nabla \phi \cdot \nabla \delta \phi +M \frac{\partial f(\phi )}{\partial \phi }\delta \varvec{\phi }+\frac{\phi -\phi ^{t-\Delta t}}{\Delta t} \delta \phi \\&-R_s c\;d\;\delta \phi ]\ dv -K_{\phi }\int _{\partial \mathcal {B}} \epsilon ^2\nabla \phi \cdot \varvec{n} \ \delta \phi \ ds\\&+K_{d}\int _{\mathcal {B}}[ d\ \delta d +l^2\nabla d \cdot \delta \nabla d +\eta _d \frac{d -d ^{t-\Delta t}}{\Delta t} \delta d \\&-\frac{\partial g(d)}{\partial d}\frac{ \Psi _{max }}{\Psi _{cri }/l}\delta d]\ dv -K_{d}\int _{\partial \mathcal {B}} l^2 \nabla d \cdot \varvec{n} \ \delta d\ ds=0, \end{aligned} \end{aligned}$$

where $\varvec{n}$ is the normal vector to the surface of the boundary. Moreover, $\varvec{t}=\varvec{\sigma }\cdot \varvec{n}$ signifies the traction (mechanical flux) applied on the boundaries. Likewise, $D \nabla c \cdot \varvec{n}$ corresponds to the nutrient flux on the boundaries. It is a common as well as justifiable assumption that the boundary term for the phase-field variable is set to zero. Thus, the flux terms $K_{\phi }\int _{\partial \mathcal {B}} \epsilon ^2\nabla \phi \cdot \varvec{n} \ \delta \varvec{\phi }\ ds$ as well as $K_{d}\int _{\partial \mathcal {B}} l^2 \nabla d \cdot \varvec{n} \ \delta d\ ds$ can be omitted. Figure 4 illustrates the boundary conditions.

The formulation of the multi-field problem (mechanical deformation, nutrient concentration, IMH and damage) in hand has been implemented via AceGen, which is an automatic differentiation (hybrid symbolic/numeric differentiation) tool. One may refer to Korelc and Wriggers (2016) for more detailed information on AceGen. Further, the generated output has been tailored to a user element using the FORTRAN programming language, which can be invoked by any FEM solver, e.g., AceFEM, ANSYS, ABAQUS. In the present work, we selected ANSYS because of its extensive pre-processor and post-processor features. The employed element is a hexahedral (brick-shaped) element, which is commonly used in FEM, with eight nodes and linear shape functions. Every node has six degrees of freedom. Three of them indicate the components of the displacement vector $\varvec{u}$. The remaining three are assigned to the nutrient concentration field *c* and the phase-field variables $\phi$ and *d*. Moreover, the variable $\alpha$ is treated as an internal variable at the Gauss points. Based on the Newton–Raphson method, an implicit iterative procedure is utilized to solve the nonlinear problem at hand. All internal and field variables are considered to be known at the previous iteration highlighted with the subscript *n*. It should be distinguished from the values of the time-dependent variables at the previously converged time step, represented using superscript $t-\Delta t$. The global system solution provides the current values for the primary variables $\varvec{u}$, *c*, $\phi$ and *d*, which are indicated by the subscript $n+1$. For the sake of simplification, the entire implemented algorithm is summarized in Table 1.

**Table 1:** Implementation algorithm in the AceGen

1. Interpolate the field variables ${\mathcal D}_I$ (components of displacement, concentration, IMH and damage) using standard linear FEM shape functions $N_I$	
	${\mathcal D}=\sum _{I}^{M} N_I{{\mathcal D}}_I, \quad {\mathcal D}_I:=u_I,v_I,w_I,c_I,\phi _I,d_I$, M: number of nodes
	$${\varvec{\mathcal D}}_e:=\bigcup _{I}^{M}{\mathcal D}_I=\left( u_1,v_1,w_1,c_1,\phi _1,d_1,...,u_{N},v_{N},w_{N},c_{N},\phi _N,d_N\right)$$
2. Initialize the Global Newton–Raphson using	
	$$\varvec{F}_{g(n+1)}=\varvec{F}_{g(n)}=(1+\alpha _n)\varvec{I}$$
	$${\varvec{F}}_{e(n+1)}={\varvec{F}}_{n+1}\cdot {\varvec{F}}^{-1}_{g(n+1)}$$
3. Compute	
	$${J}_{e(n+1)}=\text {Det}({\varvec{F}}_{e(n+1)})$$
	$${{\varvec{C}}}_{e(n+1)}=J^{-\frac{2}{3}}_{e(n+1)}{\varvec{F}}^{T}_{e(n+1)}\cdot {\varvec{F}}_{e(n+1)}$$
	$\Psi _{n+1}$ using (5)
	$${\varvec{\sigma }}_{n+1} =\frac{1}{{J}_{e(n+1)}}\frac{\partial {\Psi _{n+1}} }{\partial \varvec{F}_{e(n+1)}}\varvec{F}_{e(n+1)}^{T}$$
4. Local equation (at Gauss point): ${\mathcal R}_{\alpha }=0$	
	a. Solve the local problems, namely equation (14), at Gauss points to find the internal variables ($\alpha _{n+1}$)
	$${\mathcal R}_{\alpha }=\frac{(\alpha _{n+1}-\alpha _{n})}{(1+\alpha _{n+1})} -k_g(\phi _{n+1}-\phi _{n})$$
	b. Update ${J}_{e(n+1)}$, ${\varvec{F}}_{g(n+1)}$ and ${\varvec{F}}_{e(n+1)}$
	c. Compute $\varvec{A}=\frac{\partial \alpha _{n+1}}{\partial \varvec{\mathcal D_e}}$ which is later needed in tangent computation (step 6)
5. Compute the total weak form using (25)	
	$$\Pi =\int _{\mathcal {B}} {\varvec{\sigma }}_{n+1}:\nabla ^{sym } \delta {\varvec{u}}_{n+1}\ dv+K_c\int _{\mathcal {B}} [D \nabla c_{n+1} \cdot \nabla \delta c_{n+1}+R_c \ \delta c_{n+1}]\ dv$$
	$$+K_{\phi }\int _{\mathcal {B}} [\epsilon ^2 \nabla \phi _{n+1} \cdot \nabla \delta \phi _{n+1}+M\frac{\partial f(\phi _{n+1})}{\partial \phi _{n+1}}\delta \phi _{n+1}+\frac{\phi _{n+1} -\phi ^{t-\Delta t}}{\Delta t} \delta \phi _{n+1}$$
	$$-R_s\,c_n\,d_n\, \delta \phi _{n+1}]\ dv$$
	$$+K_{d}\int _{\mathcal {B}}[ d_{n+1}\ \delta d_{n+1} +l^2\nabla d_{n+1} \cdot \delta \nabla d_{n+1} +\eta _d \frac{d_{n+1} -d^{t-\Delta t}}{\Delta t} \delta d_{n+1}$$
	$$-\frac{\partial g(d_{n+1})}{\partial d_{n+1}}\frac{ \Psi _{max }}{\Psi _{cri }/l}\delta d_{n+1}]\ dv$$
6. Compute the residuum vector of the element	
	$${\varvec{\mathcal R}}_{e}=\frac{\partial \Pi }{\partial \delta {\varvec{{\mathcal D}}}_e}$$
7. Compute the element stiffness matrix taking into account the local internal variables	
	$${\varvec{\mathcal K}}_{e}={\frac{\partial \varvec{\mathcal R}_e}{\partial {\varvec{\mathcal D}}_e}} |_{\frac{\partial \alpha }{\partial {\varvec{\mathcal D}}_e}=\varvec{A}}$$
8. Global Newton–Raphson iteration:	
	$$(\varvec{\mathbb R},\varvec{{\mathbb D}},\varvec{\mathbb K})=\bigcup ^{all\ elements }(\varvec{\mathcal R}_e,\varvec{{\mathcal D}}_e,\varvec{\mathcal K}_e)$$
	DO WHILE $\left\| \varvec{\mathbb R} \right\| \ge \text {TOL}$ (Check the global convergence)
	Repeat steps (3) to (7)
	$${\varvec{\mathbb D}} \Leftarrow {\varvec{\mathbb D}}+\Delta {\varvec{\mathbb D}}, \quad \Delta {\varvec{\mathbb D}}=-{\varvec{\mathbb K}}^{-1}\varvec{\mathbb R}$$
	END DO
9. Go to the next time step and start from step (2)	

## Numerical examples

As stated before, the objective of this work is to show that atherosclerosis and dissection can be regarded as different pathology but with similar roots. In the former, a nutrient disruption in the artery wall due to VV dysfunction leads to an inflammatory response which results in the thickening of the artery wall, while, in the latter, an internal rupture of VVs gives rise to interlayer delamination. Hence two test cases are presented to demonstrate how these two pathological conditions can be unified from a modeling point of view.

In the first example, the strength of the artery is assumed to be infinite in order to prevent rupture (damage). In the second example, the inter-layer strength is limited to a finite value (threshold) which, when crossed, leads to inter-layer rupture. In order to test the suitability of the mathematical model and to examine the initiation of dissection (damage evolution), a 2D artery model is used for the numerical simulation. Specifically, the arterial wall’s geometry is considered a simple 2D cylinder, as shown in Fig. 4, along with the setting of boundary conditions. The material and numerical parameters required for the simulations are collected in Table 2. The material parameters concerning fracture and mechanical behavior of the coronary arteries are taken from the literature. One needs to keep in mind that there is no reference value, especially for fracture-related parameters and the literature suffers from arbitrariness and scatteredness in this regard, see e.g., Ban et al. (2021, 2022); Gültekin et al. (2019); Holzapfel et al. (2005); Badel et al. (2014).

**Fig. 5:**
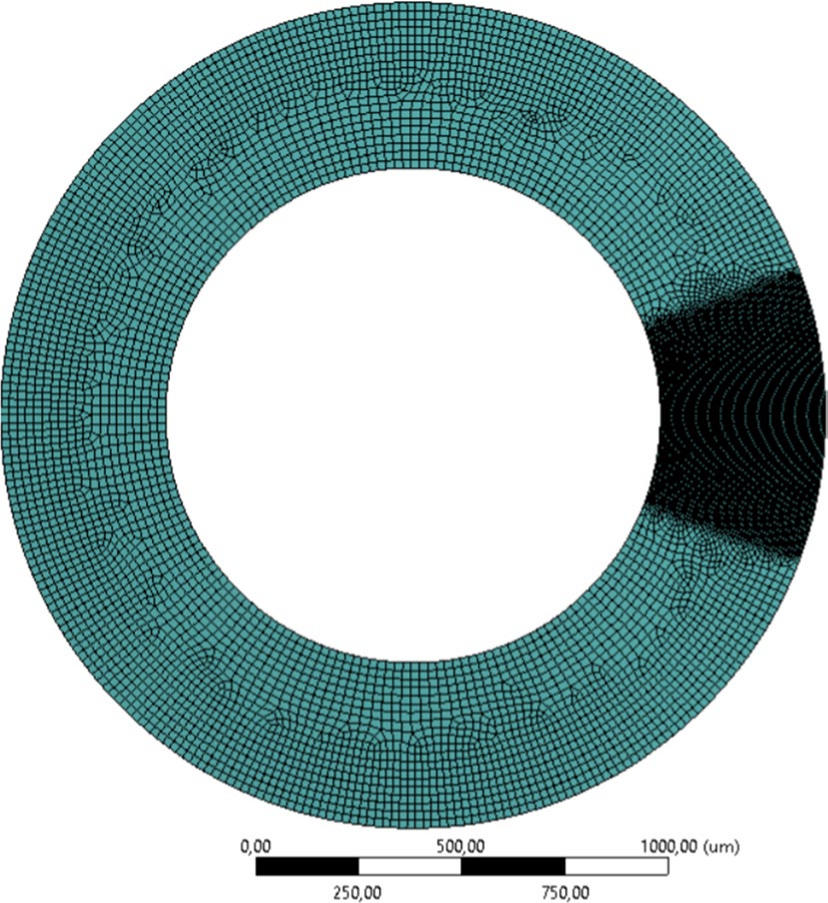
Discretized model and local mesh refinement in 2D

Although the mathematical model and numerical implementation are 3D, the numerical examples are color mainly restricted to 2D with plane strain assumption, due to computational efficiency. Furthermore, the mesh is only refined around the region where the phase-field equations are to be solved. Figure 5 shows the mesh representing the artery section Table 3.

**Table 2:** Geometrical parameters of the test cases and material constants (the values without reference are assumed by the authors)

Description	Parameter	Value	Unit	Ref
Coronary artery inner diameter	D	1200.0	${\upmu } \text {m}$	Holzapfel et al. (2005)
Coronary artery wall thickness	t	400.0	$\upmu \text {m}$	Holzapfel et al. (2005)
Initial occlusion location	S	30.0	$\upmu \text {m}$	
Overgrowth constant	$k_g$	10	$Time ^{-1}$	
Max. internal pressure	$p_{max }$	$120 \ (\approx 16)$	mmHg (kPa)	Ramanathan and Skinner (2005)
Cell consumption	$R_c$	$10^{-2}$	$\upmu \text {g} \, \upmu \text {m} ^ {-3} \, Time ^{-1}$	
Max. diffusion coefficient	$D_{max }$	$10^{3}$	$\upmu \text {m}^2 \, Time ^{-1}$	
Min. diffusion coefficient	$D_{min }$	1.0	$\upmu \text {m}^2 \, Time ^{-1}$	
Hematoma rate	$R_s$	100	$\upmu \text {m} \, Time ^{-1}$	
Shear modulus	$\mu$	$\sim 30$	kPa	Holzapfel et al. (2005)
Poisson’s ratio	$\nu$	0.49	–	
Inter-layer fracture energy	$\frac{\Psi _{cri }}{l}$	100	$kPa$	Gültekin et al. (2019)
Damage length scale	*l*	2 h	$\upmu \text {m}$	
Damage viscosity parameter	$\eta _{d}$	0.01	–	
Numerical minimum allowed damage	$d_{min }$	$10^{-4}$	–	
Phase-field parameter	$\epsilon$	25	–	
Phase-field parameter	*M*	1	–	
Numerical parameter	$K_{\phi }$	1	–	
Numerical parameter	$K_c$	1	–	
Numerical parameter	$K_d$	1	–	
Mesh size 2D, coarse/fine	h	20/2	$\upmu \text {m}$	
Mesh size 3D, coarse/fine	h	40/10	$\upmu \text {m}$

**Table 3:** Geometrical parameters of VV tree fractal (the values without reference are assumed by the authors)

Description	Parameter	Value	Unit	Ref
Tree trunk	$L_0$	80	$\upmu \text {m}$	
Second branching parameter	$\lambda _2$	1.0	–	
Third branching parameter	$\lambda _3$	1.0	–	
Forth branching parameter	$\lambda _4$	1.0	–	
Second branch angle 2D	$\gamma _2$	$\frac{2\pi }{3}$	Rad	
Third branch angle 2D	$\gamma _3$	$\frac{2\pi }{3}$	Rad	
Forth branch angle 2D	$\gamma _4$	$\frac{2\pi }{3}$	Rad	

### Inflammatory response of artery wall without dissection

**Fig. 6:**
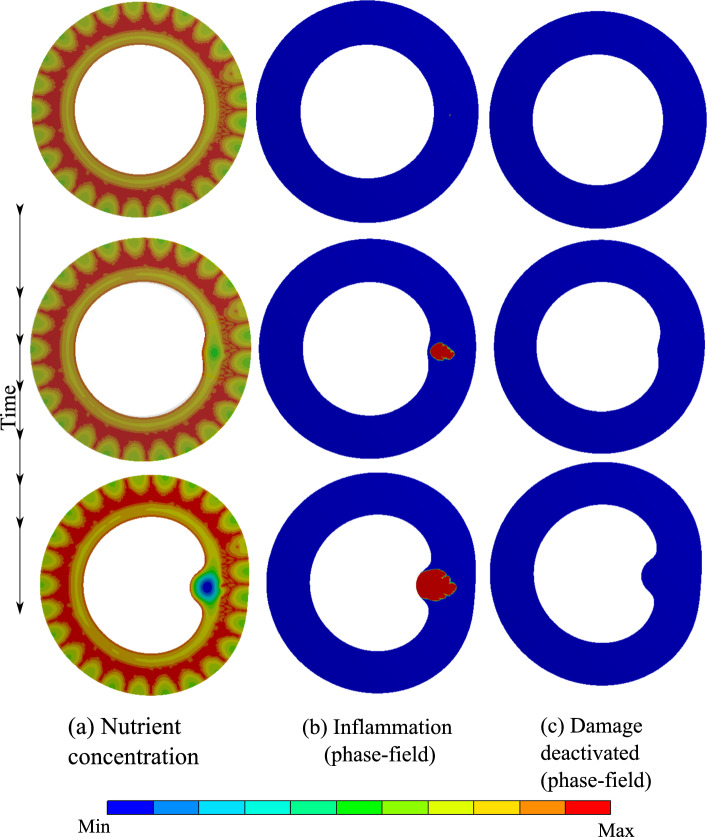
2D atherosclerosis (without damage evolution, i.e., $d=0$): variation of dimensionless nutrient and inflammation in the course of time

The first test case predicts atherosclerosis, which is wall thickening due to the malfunction of the nourishment network namely VVs. The VV network supplies the wall with the nutrient as an auxiliary mechanism to the diffusion from the lumen. The presence of VVs substantially changes the nutrient distribution compared to simple diffusion-dominated nourishment from the lumen. Based on the presented mathematical model, inflammation development is regulated by the nutrient gradient. Figure 6 depicts the progression of the inflammation in the presence of VVs. One can see that the phase-field approach is capable of capturing this phenomenon. The wall swells and an inner bulge is generated giving rise to atherosclerosis. From a modeling point of view, to avoid the appearance of damage (rupture) in our general multi-physics model, a very large value is chosen for the critical fracture energy so that the damage mechanism is not activated despite very large deformation.

**Fig. 7:**
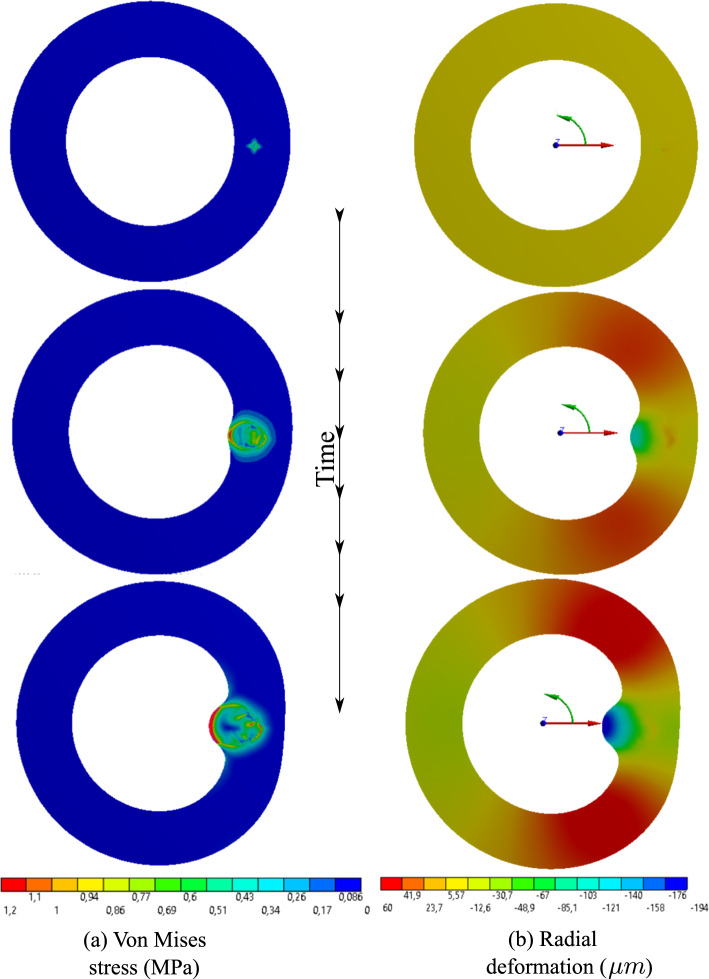
2D atherosclerosis (without damage evolution, i.e., $d=0$): distribution of von Mises stress and the radial deformation in the course of time

**Fig. 8:**
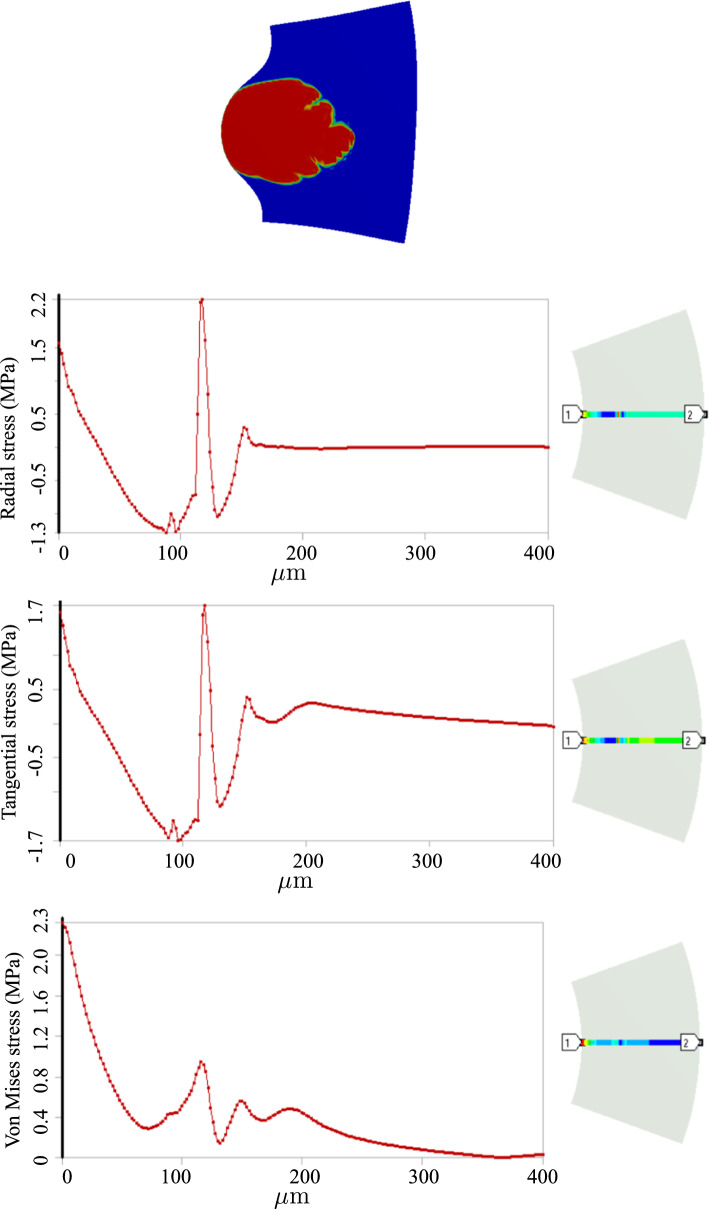
2D atherosclerosis (without damage evolution, i.e., $d=0$): variation of stresses across the artery thickness

Figure 7 shows the evolution of equivalent stress as well as the radial deformation. It illustrates that the inflammatory region is highly under stress. A closer look at the radial and tangential stresses in Fig. 8 reveals that the inflammatory zone is predominantly subjected to compressive stresses while the tissue surrounding bears tensile stresses. That means the stress gradient is significant across the boundaries of inflammation.

### IMH-driven response of artery wall with dissection

**Fig. 9:**
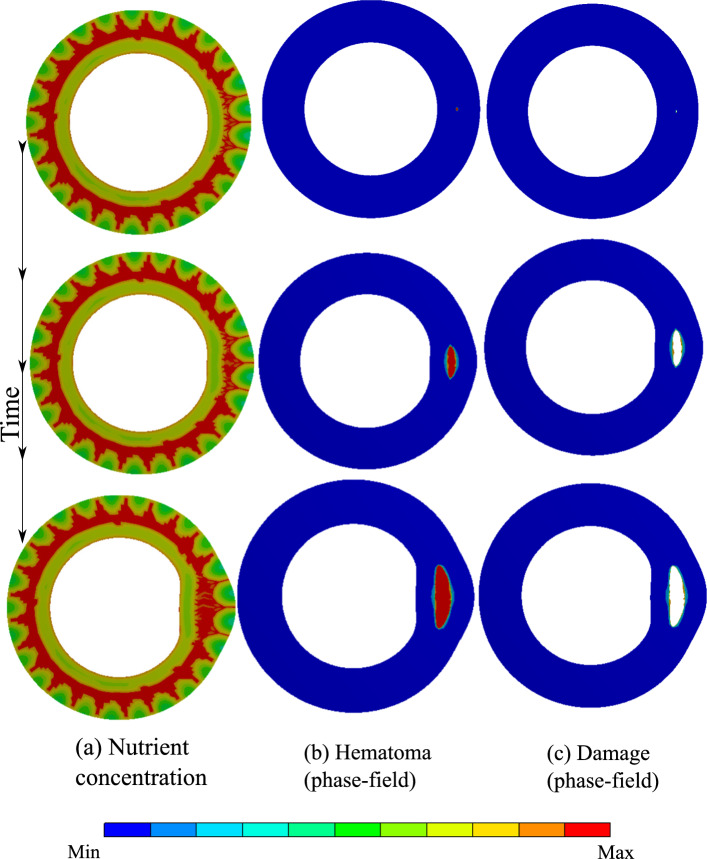
2D dissection (with damage evolution, i.e., $d\ne 0$): variation of dimensionless nutrient and hematoma in the course of time

The dissection example is, in principle, identical to the previous one except for the damage mechanism being activated. It is achieved by setting the inter-layer fracture energy $\Psi _{cri }$ to a finite value, see Table 1. As a result, rupture occurs when the fracture threshold is exceeded. Here the IMH is driven by the seepage of blood from the VVs to the aperture and consequently contributes to the propagation of the ruptured region, see Fig. 9. As stated before, the progression of the interlayer debonding is similar to the hydraulic fracture (hydrofracking) phenomenon in which pressurized fluid is utilized to fracture the bedrock Mauthe and Miehe (2017).

The interlayer debonding may extend to a very long distance (axial extension) and cause acute conditions that might be life-threatening. A well-known example of such a scenario is the aortic dissection in which the inner layer of the wall is torn and the blood flows between the layers of the aortic wall instead of the lumen. It leads to a profound drop in blood pressure and quick death. Here, the long-term and large-scale propagation of the ruptured region is not considered in the simulation. Rather, the early phases of the rupture are investigated and hence the nucleation of the micro-injury within the wall and its propagation at the earlier phases is of interest. The full fluid-solid interaction analysis of the pathology in the later stages necessitates a fluid flow analysis in which the blood penetrates the ruptured region from the lumen and expands it. The full analysis is left for future works.

**Fig. 10:**
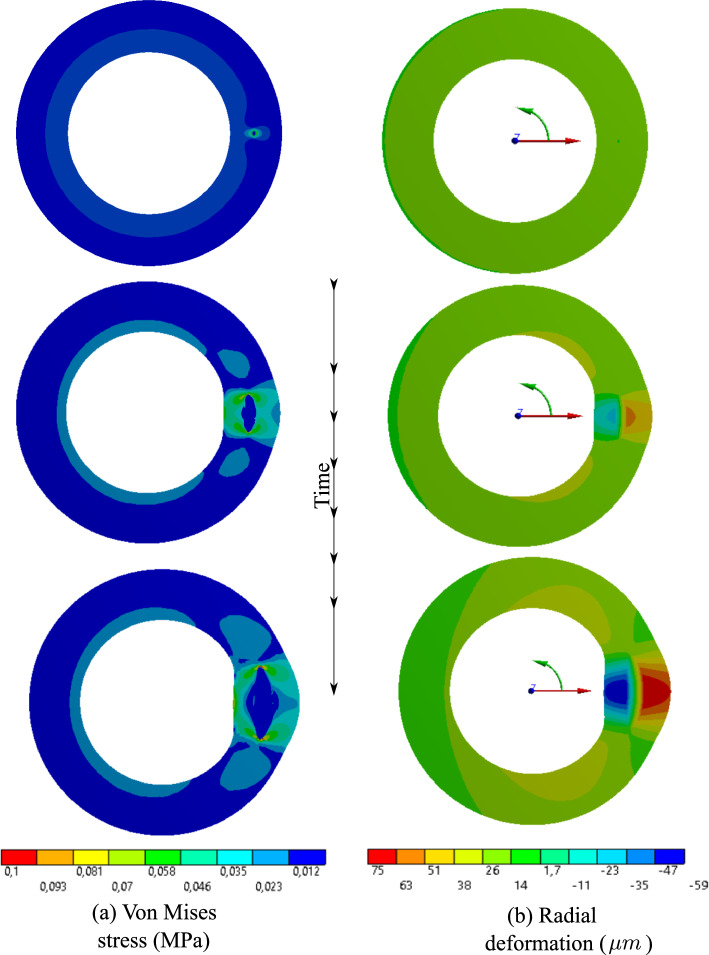
2D dissection (with damage evolution, i.e., $d\ne 0$): distribution of von Mises stress and the radial deformation in the course of time

A comparison of the mechanical quantities in atherosclerosis and dissection scenarios is quite informative. In this regard, Fig. 10 can be compared with Fig. 7. The two phenomena lead to substantially different responses of the artery wall. While the integrity of the artery wall is kept intact in the case of atherosclerosis, the rupture of the wall is the main manifestation of dissection. From a mathematical point of view, the former ensures a continuous profile of physical quantities such as stresses and displacements, but the latter results in a jump in the profile of field variables due to the presence of delamination. Figures 10 and 7 depict the variation of stresses and displacements across the thickness of the wall in two cases. The ruptured region cannot sustain mechanical loads and hence the state of stress inside the aperture is hydrostatic compression which can be interpreted as the pressurized blood. The crack tip, however, is highly under stress as expected.

**Fig. 11:**
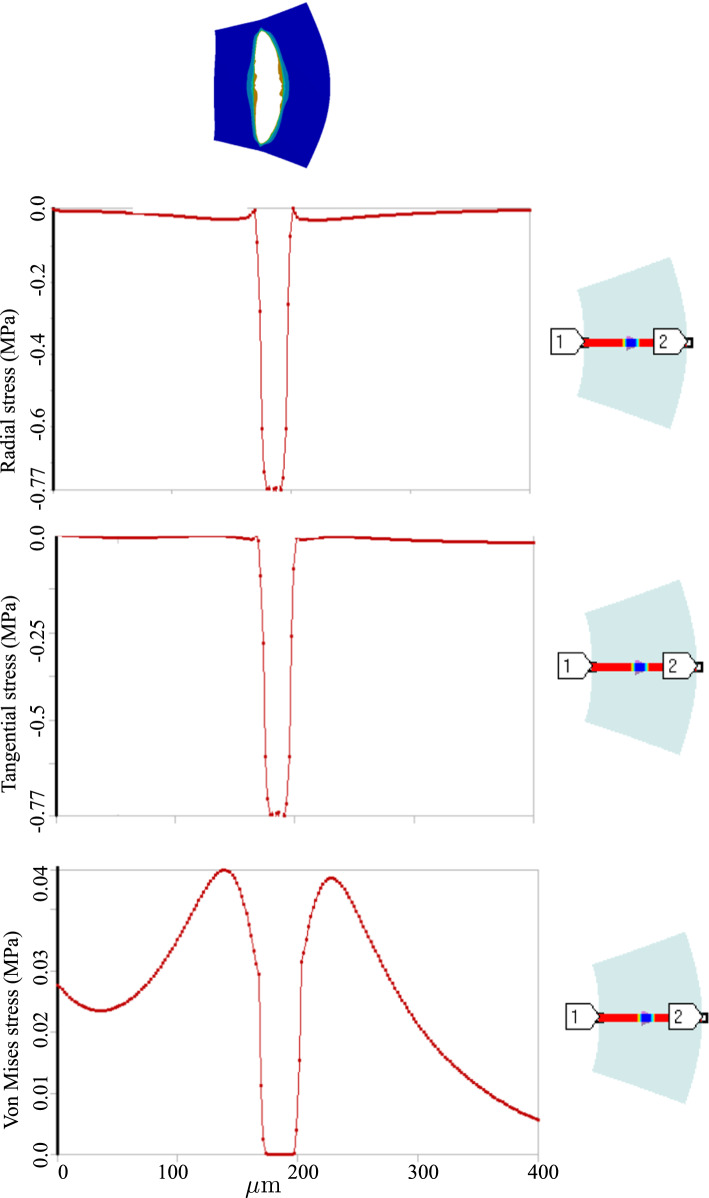
2D dissection (with damage evolution, i.e., $d\ne 0$): variation of stresses along the defined path from point 1 to point 2

One can realize more clearly the distinction between stress states in two cases by looking at the radial and tangential stress profiles in Figs. 8 and 11. Unlike the fractured zone (Fig. 11), the inflammatory zone (Fig. 8) undergoes intense mechanical stresses (because there is no softening mechanism in the model) with a very sharp gradient at the interface between the inflammation and the surrounding tissues. Nonetheless, in Fig. 11, the fractured zone bears a compressive and almost isotropic state of stress (due to pressurized blood in the ruptured zone) which is reflected in small von Mises stress. Moreover, the stress concentration is also observed at the crack tips (relatively high von Mises stress at the crack tip).

**Fig. 12:**
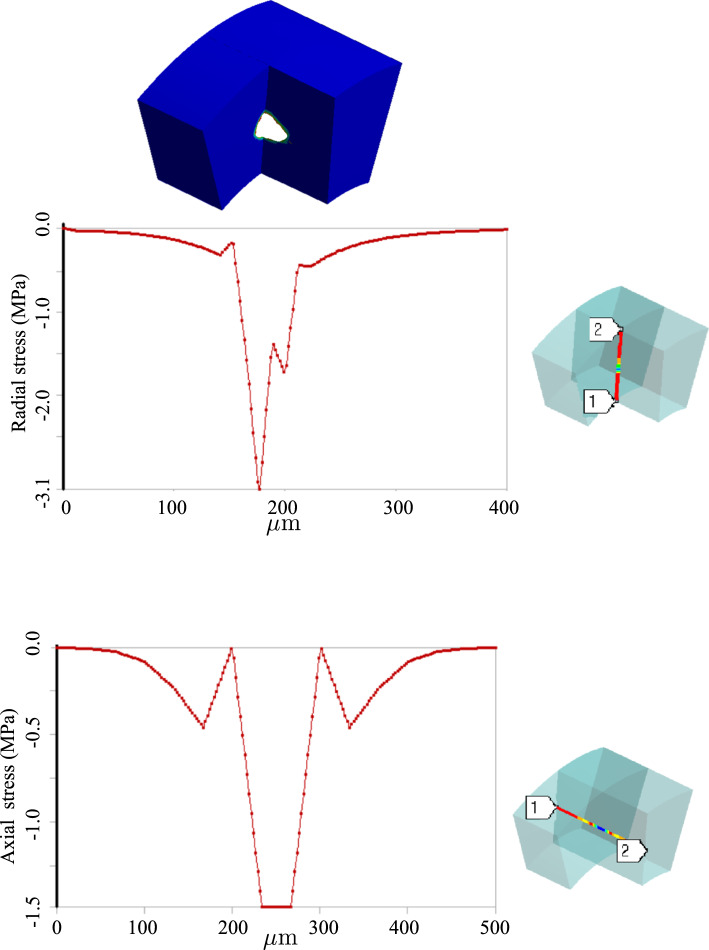
3D dissection (with damage evolution, i.e., $d\ne 0$): variation of stresses along the defined path from point 1 to point 2

**Fig. 13:**
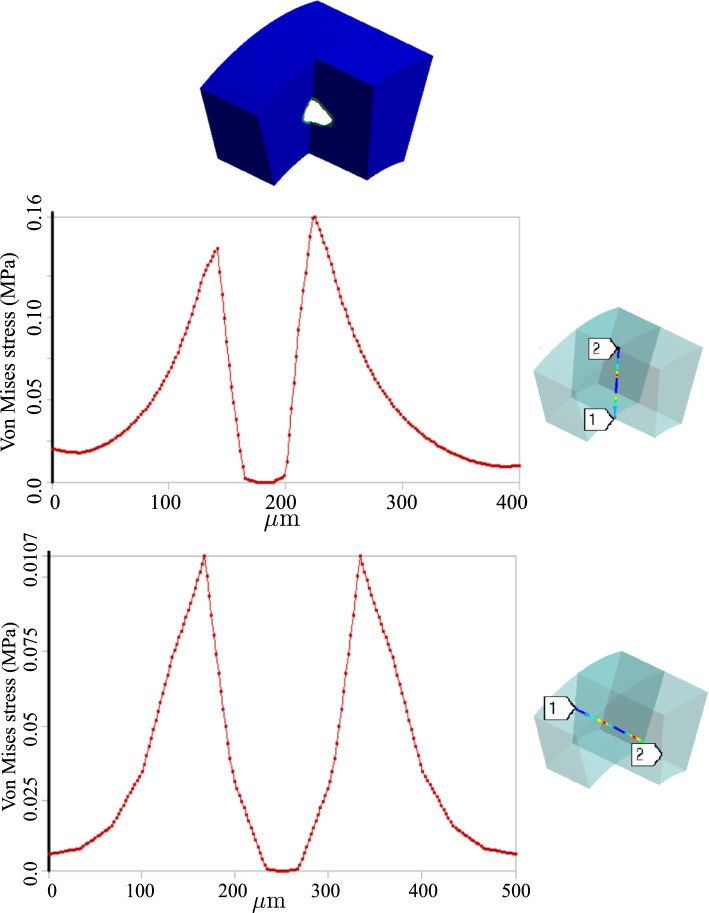
3D dissection (with damage evolution, i.e., $d\ne 0$): variation of stresses along the defined path from point 1 to point 2

Inflammation and dissection differ also in the way that they induce deformation in the artery wall. Different deformation modes are observed when one compares the radial deformation in Figs. 7 and 10. A jump in radial deformation and in the case of dissection clearly shows the discontinuity of geometry. However, as stated before, the dissection expands in both tangential and axial directions. To capture the axial propagation of the ruptured zone, a 3D model is inevitable and a 2D histological representation is not sufficient. However, the computational cost of 3D cases is drastically higher than that of 2D cases. Here the total number of degrees of freedom for 2D examples is roughly $1.5\times 10^5$ while it exceeds $4\times 10^6$ in 3D. Nonetheless, using a coarser mesh, a 3D test case is also executed to examine the axial variation of stress components around the regions undergoing dissection. Figs. 12 and 13 illustrate the profile of stress components along radial and axial paths defined in the region of interest. One can realize that similar to 2D examples, the ruptured region filled with blood bears high compressive and almost hydrostatic stresses (very small von Mises stress), while the crack tips undergo relatively high von Mises stresses.

## Conclusion

In this work, a mathematical model was presented for the prediction of the dissection initiation in the arteries. The mathematical framework is an extension of the model developed for atherosclerosis in Soleimani et al. (2021). By using the extended model for the prediction of atherosclerosis and dissection, this work advocates the idea of a unified framework proposed by the fifth author that explains different arterial diseases, namely atherosclerosis, aneurysm and dissection, using a unified approach. There are some limitations that this paper is based on. They can be addressed in order to improve the model.

One direction for the extension of this work is clinical validation. The outcome of the computational models can be validated against clinical medical data. For example, in the case of aortic dissection, high-resolution CT or MRI images are pretty usable Ko et al. (2021); Murillo et al. (2021).

The constitutive modeling of the artery has also a potential for improvement. The assumed uniform wall can be replaced by a multi-layer structure each of whose layers has different mechanical properties due to the presence of reinforcing fibers.

Moreover, the fluid flow in the lumen can be coupled with the existing model in order to capture the propagation of the dissection on larger scales. This extension necessitates conducting an affordable 3D analysis using parallel solvers. The reason is that resolving the phase-field variables as well as the VV network in 3D needs a sufficiently fine mesh. It leads naturally to millions of degrees of freedom for such a nonlinear and coupled multiphysics problem.

## Data Availability

Raw data and the developed ANSYS user element are available from the corresponding author on request.
